# Dr. Hiram Parker Trull

**Published:** 1915-10

**Authors:** 


**Dr. Hiram Parker Trull,** Buffalo 1868, died at his home in Williamsville, N. Y., September 14, aged 72. He had been an invalid for several years.

